# Hippo Pathway and Skeletal Muscle Mass Regulation in Mammals: A Controversial Relationship

**DOI:** 10.3389/fphys.2017.00190

**Published:** 2017-03-29

**Authors:** Olouyomi Gnimassou, Marc Francaux, Louise Deldicque

**Affiliations:** Institute of Neuroscience, Université catholique de LouvainLouvain-la-Neuve, Belgium

**Keywords:** Yap, Taz, growth, size, mTOR

## Abstract

Skeletal muscle mass reflects a dynamic turnover between net protein synthesis and degradation. In addition, satellite cell inclusion may contribute to increase muscle mass while fiber loss results in a reduction of muscle mass. Since 2010, a few studies looked at the involvement of the newly discovered Hippo pathway in the regulation of muscle mass. In line with its roles in other organs, it has been hypothesized that the Hippo pathway could play a role in different regulatory mechanisms in skeletal muscle as well, namely proliferation and renewal of satellite cells, differentiation, death, and growth of myogenic cells. While the Hippo components have been identified in skeletal muscle, their role in muscle mass regulation has been less investigated and conflicting results have been reported. Indeed, the first studies described both atrophic and hypertrophic roles of the Hippo pathway and its effectors Yap/Taz using different biochemical approaches. Further, investigation is therefore warranted to determine the role of the Hippo pathway in the regulation of skeletal muscle mass. New components of the pathway will probably emerge and unsuspected roles will likely be discovered due to its numerous interactions with different cellular processes. This mini-review aims to summarize the current literature concerning the roles of the Hippo pathway in the regulation of muscle mass and to develop the hypothesis that this pathway could contribute to muscle mass adaptation after exercise.

## Introduction

Skeletal muscle mass reflects a dynamic turnover between net protein synthesis and degradation. In addition, satellite cell inclusion may contribute to increase muscle mass while fiber loss results in a reduction of muscle mass. At the molecular level, skeletal muscle mass regulation is the result of coordinated activation/inhibition of numerous signaling pathways. The first identified signaling pathways involved in organ genesis and development were, non-exhaustively, Notch, Wnt, transforming growth factor-beta (TGF-β), and Hedgehog (Pan, 2010). The last decades, the mammalian target of rapamycin (mTOR) and Hippo pathways were found to be additional regulators of organs size (Csibi and Blenis, 2012). Contrary to the mTOR pathway, which is relatively well-described, numerous mechanisms of regulation of the Hippo pathway remain unclear. Indeed, in Drosophila, after identification of Warts (Wts) in the 1990s, Salvador (Sav) and Hippo in the 2000s, a first description of the Hippo scaffolded pathway was made (Pan, 2010). First identified in mouse, and then in human, some mammalian homologs of the components of the Hippo pathway in Drosophila are thought to be involved in organ size regulation, and many studies began to focus on the Hippo pathway in mammals. The canonical Hippo pathway is now described in mammals but crosstalks and interplays with other signaling pathways as well as its role in organ size regulation are still unclear (Yu et al., 2015).

Recently, the Hippo pathway has been of great interest for deep comprehension of molecular mechanisms underlying etiology, physiopathology and treatment of pathologic features in chronic diseases like cancers, diabetes, chronic obstructive pulmonary disease, or renal failure. Unfortunately, its involvement in exercise physiology and muscle mass regulation in non-pathological states has been under-investigated up to now (Watt et al., 2015). Therefore, this mini-review aims to provide a focused highlight on the Hippo pathway organization, regulation, and involvement in skeletal muscle mass regulation in mammals and to develop the hypothesis that this pathway could contribute to muscle mass adaptation after exercise.

## Organization of the hippo pathway

### Core components: Mst1/2 and Lats1/2

**Figure 1:**
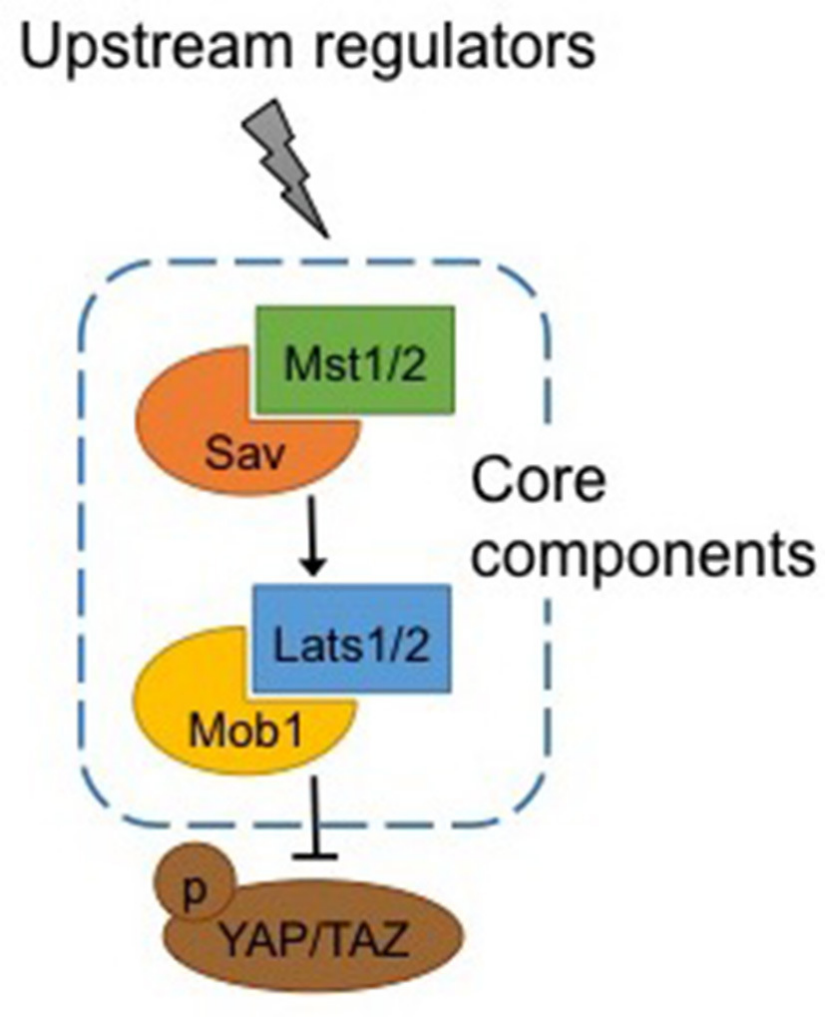
**Canonical Hippo pathway**. Core components of the Hippo pathway in mammals. Mst1/2 is phosphorylated by upstream regulators and binding with Sav enhances its affinity for Lats1/2, which in turn binds with Mob1. Activation of Mst1/2 and Lats1/2 results in phosphorylation and inactivation of Yap/Taz. See text for abbreviations.

Warts (Wts), core components of the Hippo pathway identified in Drosophila, and their homologs in mammals, large tumor suppressor kinases 1 and 2 (Lats1/2), are protein kinases of the nuclear Dbf2-related (NDR) family (Figure 1). Lats1/2 are activated by phosphorylation by macrophage stimulating proteins 1/2 (Mst1/2), members of the Ste20 protein kinase family (Qin et al., 2013). By binding to Lats1/2, Mob1 enhances the affinity of the latter to Mst1/2. Mst1/2 are themselves phosphorylated by upstream activators, and binding with the adaptor protein Sav enhances their affinity with Lats1/2 (Qin et al., 2013).

### Yap and taz are phosphorylated and inhibited by the hippo pathway

**Figure 2:**
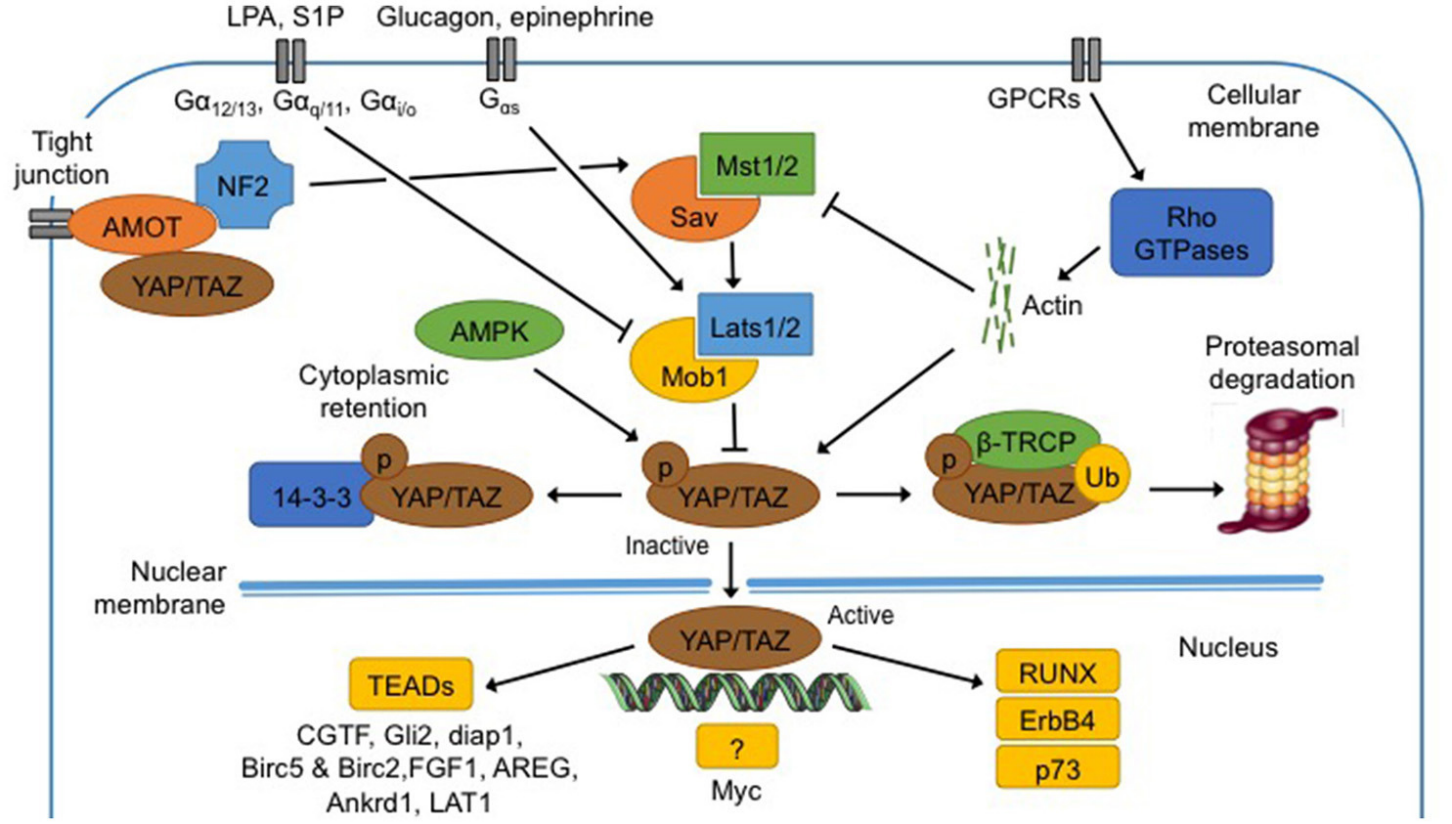
**Regulation and functions of the Hippo pathway**. Hippo pathway core components, Mst1/2 and Lats1/2, are regulated by membrane protein (NF2, AMOT), membrane receptors (GPCR and downstream effectors Rho GTPases), metabolites (epinephrine, glucagon, LPA and S1P), low ATP levels and extracellular matrix. When phosphorylated (1) at S127 (S89 for Taz), Yap is bound to 14-3-3 protein for cytoplasmic retention; (2) at S381 (S311 for Taz), β-TRCP is recruited and Yap/Taz is ubiquitinated for proteasomal degradation. In the non-phosphorylated state, Yap translocates into the nucleus, and binds to TEAD proteins to induce transcription of several genes (CGTF, Gli2, diap1, Birc5, Birc2, FGF1, AREG, Ankrd1, and LAT1). Yap can bind an unknown transcription factor to induce transcription of Myc. Finally Yap can directly bind proteins like RUNX, ErbB4, and p73. See text for abbreviations.

Yes-Associated Protein (Yap) and its ortholog Transcriptional co-activator with PDZ binding motif (Taz) are the main effectors of the Hippo pathway. The critical event is the phosphorylation of those effectors by Lats1/2 (Piccolo et al., 2014; Figure 2).

#### Phosphorylation and cytoplasmic retention

Yap contains five HXRXXS sequences that can be phosphorylated by Lats kinases while Taz contains four of them. Phosphorylation of Yap at S127 and of Taz at S89 leads to binding with 14-3-3 protein in the cytoplasm, maintaining Yap/Taz in the cytoplasm (Zhao et al., 2011).

#### Non-phosphorylation-dependent cytoplasmic retention

Yap/Taz can also be inhibited and maintained close to cellular membrane by protein-protein bindings with the Angiomotin's (AMOT) protein family (Zhao et al., 2011).

#### Phosphorylation and degradation

When Yap is phosphorylated at S381, and Taz at S311 by Lats1/2, casein kinase 1 (CK1δ/ε) can in turn phosphorylate Yap at S400 and S403 and Taz at S314 (Zhao et al., 2011). This induces the formation of a phosphodegron, the function of which is to recruit β-transducin repeat-containing protein (β-TRCP) E3 ubiquitin ligase. The formation of phosphodegron, in association with activation of β-TRCP, leads to Yap/Taz degradation by the proteasome in the cytoplasm (Kodaka and Hata, 2015).

In summary, activation of the Hippo pathway induces Yap/Taz phosphorylation and inhibition of the latter complex, which results in its sequestration in the cytoplasm and subsequent degradation by the proteasome.

## Upstream regulators of the hippo pathway

Numerous cues, amongst which metabolites, nutrients and mechanical signals, are thought to regulate the Hippo pathway, even if the molecular mechanisms are still unclear (Fischer et al., 2016). In mammals, neurofibromatosis 2 (NF2) mutations induce benign tumors while those tumors are repressed by Yap suppression. It has therefore been postulated that NF2 could be an upstream regulator of Hippo and Yap (Benhamouche et al., 2010). Recent studies showed that AMOT can bind NF2 directly, promoting interaction and activation of Mst1/2 and sequestration of Yap/Taz in the cytoplasm (Li et al., 2015). All together, regulation of Yap activation by NF2 is clearly more complex than it was thought.

G-protein-coupled-receptors (GPCRs), a vast family of membrane receptors, are identified as regulators of the Hippo pathway and Yap/Taz (Santinon et al., 2016). The regulation by GPCRs may activate or inhibit the Hippo pathway and actin filaments and Rho GTPases are thought to mediate the interaction between GPCRs and Yap/Taz (Yu et al., 2012). The Hippo pathway is also differentially regulated according to the ligands that bind to the GPCRs, amongst which several extracellular metabolites (Santinon et al., 2016). The first metabolite discovered was mevalonate that is responsible for acetyl-CoA conversion in isoprenoides precursors such as cholesterol, biliary acid or steroids, which can all activate the Hippo pathway (Santinon et al., 2016). Similarly, epinephrine and glucagon, mediated by Gαs-coupled signals, activate Lats1/2 and consequently inhibit Yap/Taz. On the contrary, lysophosphatidic acid (LPA) and sphingosine 1-phosphate (S1P), mediated by Gα12/13-, Gαq/11-, and Gαi/o-coupled signals, repress Lats1/2 (Santinon et al., 2016).

The AMP-activated protein kinase (AMPK) pathway is implicated in cellular homeostasis and can inhibit the Hippo pathway (Wang et al., 2015a). In energy stress conditions, i.e low levels of ATP, AMPK promotes the inhibitory activity of tuberous sclerosis complex (TSC1-TSC2), an upstream component of the mTOR pathway. AMPK also phosphorylates Yap at S94 and S61 and maintains the latter in the cytoplasm (Wang et al., 2015a). These two mechanisms trigger catabolic pathways aiming to recover energy homeostasis and basal AMPK activity (Santinon et al., 2016).

Finally, the Hippo pathway is regulated by cell-cell contact and extracellular matrix. Indeed, Yap is phosphorylated and maintained in the cytoplasm in densely populated cells, while lysis of cell-cell junctions induces Yap/Taz translocation to the nucleus. Therefore, an overexpression of Yap in the nucleus is observed in sparsely populated cells (Low et al., 2014).

## Downstream effectors of the hippo pathway

Yap/Taz are co-activators and as such, do not possess DNA binding sites. To induce their transcriptional program, Yap/Taz have to bind to transcription factors such as to the TEADs family proteins (Tumaneng et al., 2012; Chaillou et al., 2015; Hansen et al., 2015). Yap and TEADs together can induce the transcription of specific genes such as connective tissue growth factor (CTGF), Gli2, mammalian homolog of Diap1 and baculoviral IAP repeat-containing 5 and 2 (Birc5 and Birc2), ankyrin repeat domain 1 (Ankrd1), and L-type amino acid transporter 1 (LAT1). Yap can bind an unknown transcription factor to induce transcription of Myc (Tumaneng et al., 2012). Finally, Yap can bind other proteins like runt-related transcription factor (RUNX), ErbB4 and p73 but none of those complexes seems to regulate organ size or development (Zhao et al., 2011).

Yap/Taz, and more broadly the Hippo pathway, are involved in many interactions and crosstalks with other signaling pathways to regulate cell cycle and cell fate (Fischer et al., 2016). These interactions and crosstalks are described in the next section.

## Interplay with other signaling pathways

### Hippo and transforming growth factor-β pathways

TGF-β plays an important role in the proliferation of stem cells and tumor genesis via Smads activation as effectors (Saito and Nagase, 2015). When phosphorylated, Smad1 affinity to Yap is enhanced, which in turns activates proliferation and renewal of embryonic stem cells (Grannas et al., 2015). Similarly, Taz, and Smad2/3 binding induces their nuclear localization, transcription of TGF-β targets genes, and renewal of embryonic stem cells. However, the exact nature of interactions between Yap/Taz and Smads and their contribution to organ size regulation remain unclear (Saito and Nagase, 2015).

### Hippo and Wnt pathways

Wnt is decisive in stem cell proliferation, differentiation and fate, the aberrant stimulation of which induces tumors genesis (Hergovich and Hemmings, 2010). Stimulation of Wnt receptors by their ligands elicits a disheveled (Dvl)-dependent inhibition of β-catenin complexes degradation, β-catenin accumulation in the nucleus and interaction with T-cell factor/Lymphoid enhancer factor (TCF/Lef) and finally transcription of Wnt-regulated genes. Contrarily to Wnt, Taz inhibits Dvl1, 2 and 3 by binding and retaining β-catenin in the cytoplasm, preventing the latter to exert its transcriptional activity (Imajo et al., 2012). Seeing that Mst1/2 and Lats1/2 can phosphorylate Taz, they are thought to be able to repress Wnt as well (Kim and Jho, 2014). In summary, the Hippo pathway can repress Wnt signaling by blocking Dvl or maintaining β-catenin in the cytoplasm, however, the contribution of the interaction between the Hippo and Wnt pathways to organ size regulation remains unknown (Xu et al., 2014).

### Hippo and sonic-hedgehog pathways

The Sonic-Hedgehog pathway is the main regulator of cell differentiation, proliferation and polarity (Zhao et al., 2010). In medulloblastoma elicited by excessive Sonic-Hedgehog and Wnt activation, an overexpression of Yap and TEAD1 is observed (Fernandez et al., 2009). Moreover, Yap and TEAD1 binding may induce expression of Gli2, a Sonic-Hedgehog effector, which re-enforces the hypothesis of an interaction between Hippo and Hedgehog. However, it remains unknown whether other components of the Hippo pathway are involved in the interaction with the Sonic-Hedgehog pathway (Zhao et al., 2010).

### Hippo and Akt-mTOR pathways

The Hippo and the Akt-mTOR pathways are responsible for the regulation of cell numbers and cell size, respectively. mTOR regulates cell size in response to nutrients and growth factors (Lee et al., 2007). Hippo regulates cell number by phosphorylating Yap/Taz, which in turn will result in a repression of cell proliferation and a stimulation of apoptosis in mammals (Csibi and Blenis, 2012). An overexpression of the effectors of Hippo, i.e., Yap/Taz, increases the phosphorylation of Akt and S6 Kinase1 (S6K1). Inversely, Yap is maintained in the cytoplasm by 14-3-3 protein and its transcription repressed when phosphorylated by Akt (Kodaka and Hata, 2015). On the other hand, Yap has been shown to promote skeletal muscle hypertrophy independently of any change in mTOR activity (Watt et al., 2015). In summary, while it is clear that the Hippo and Akt-mTOR pathways may interact, the direction and the consequences of the interaction do not seem straightforward.

## Hippo pathway and regulation of organ size

### Hippo and organ size control

The role of the Hippo pathway in organ size management has been convincingly demonstrated by studies in mice. Specific alteration of Mst1/2, Lats1/2, or Sav in liver cells provokes up to a four-fold growth (Zhao et al., 2011). In another study, the transcriptional activity of Yap was increased during regrowth after partial liver ablation while at the same time, Mst1/2 and Lats1/2 were inhibited (Grijalva et al., 2014). This higher activity of Yap was quasi-automatically repressed at the end of regrowth and Mst1/2 and Lats1/2 inhibition released (Grijalva et al., 2014). The situation is more complex in the pancreas as Hippo pathway activity seems to be compartment-dependent. Indeed, Mst1/2 inhibition induced hyperplasia and cell death in the exocrine compartment while there was no incidence in the endocrine compartment (George et al., 2012). In the intestine, Yap ablation did not repress development but limited post-lesion regrowth (Cai et al., 2010). All together, those studies show the role of the Hippo pathway in the regulation of organ size.

### Hippo and stem cells

The Hippo pathway is thought to manage stem cell regulation, renewal, and expansion. Indeed, Yap is activated in satellite cells and inhibited during differentiation of embryonic stem cells. Furthermore, embryonic stem cells stay quiescent under regulation by Taz (Hiemer and Varelas, 2013). In intestinal progenitors, hepatic oval cells, neural and epidermal stem cells, Yap is generally required for their stemness, promoting proliferation but inhibiting differentiation (Judson et al., 2012).

## Hippo pathway and skeletal muscle mass regulation

The Hippo components have been identified in myogenic cells *in vitro* and in skeletal muscle *in vivo* (Watt et al., 2010) but their role in muscle mass regulation is less investigated. Given its multiple interactions with other signaling pathways, it is postulated that the Hippo pathway could play a role in different regulatory mechanisms, namely proliferation and renewal of satellite cells, differentiation, death and growth of myogenic cells (Yu and Guan, 2013).

### Role of the hippo pathway during myogenesis

As observed in other tissues, the mRNA and protein expression of Yap were high during myogenic cell proliferation and low during differentiation (Watt et al., 2010; Judson et al., 2012). In addition, during C2C12 cell proliferation, Yap Ser127 phosphorylation was low and Yap localized to nuclei. Upon differentiation, Yap Ser127 phosphorylation increased about 20-fold and Yap translocated from the nucleus to the cytosol. During the differentiation phase, overexpression of hYap1 S127A, a mutant form of Yap which cannot be phosphorylated at S127 by Lats1/2, induced a decrease in myogenin and Mef2c expression, two differentiation genes, and a persistent expression of Myf5, a myoblast proliferation gene (Watt et al., 2010). Those results are consistent with the hypothesis that phosphorylation of Yap at S127 is necessary for myoblasts to withdraw the cell cycle and to differentiate (Watt et al., 2010). Similar results were obtained in satellite cells isolated from mouse muscle (Judson et al., 2012). In that study, constitutive Yap activity expanded the pool of activated satellite cells and satellite cell-derived myoblasts but prevented their differentiation. Microarray analyses identified regulators of the cell cycle, ribosomal biogenesis and modulators of myogenic differentiation as genes targeted by Yap. Yap was found to bind TEAD and co-activate muscle-specific cytidine-adenosine-thymidine (MCAT)-elements in myoblasts. In another study in postnatal myogenic cells of mice, a high expression of Mst1/2 was found followed by a decrease within the first 3 weeks of life (Wei et al., 2013). All together, those results suggest a role of the Hippo pathway in *in vitro* myogenesis as well as in postnatal muscle development *in vivo*.

### Role of the hippo pathway in mouse skeletal muscle

Due to its regulatory role in the determination of cell size in general, it can logically be expected that the Hippo pathway regulates muscle cell growth as well. To investigate this hypothesis, an inducible skeletal muscle fiber-specific knock-in mouse model (MCK-tTA-hYAP1 S127A) was developed to test whether the over expression of constitutively active Yap (hYAP1 S127A) is sufficient to stimulate muscle hypertrophy or changes in fiber type composition (Judson et al., 2013). Unexpectedly, expression of this mutant form of Yap caused, ~5 weeks later, a kind of myopathy together with muscle atrophy, which was followed by a regenerative process comprised of activation, proliferation and differentiation of satellite cells. At a molecular level, Myf5, myogenin, and Pax7, markers of satellite cells activation and differentiation, were highly expressed at the mRNA level as well as Atrogin-1 and MuRF-1, two markers of protein degradation. Of note, fiber type composition remained unchanged and the processes of atrophy and degeneration remained reversible when preventing the expression of the mutant form of Yap (Judson et al., 2013).

Contrary to the previous study, overexpressing total Yap, instead of active Yap, could induce hypertrophy via interaction with TEADs and independently of any activation of mTOR (Watt et al., 2015). It was also found that total Yap expression and phosphorylation at S112 decreased during maturation of the myofibrils (Watt et al., 2015). Furthermore, repression of Yap expression caused a reduction in the size of myofibrils, and ultimately a decrease in muscle mass. This reduction in mass was associated with slightly increased, though not significant, expression of the markers of protein degradation Atrogin-1, Musa1, and MuRF-1 (Watt et al., 2015). It was therefore concluded that the expression of Yap would be necessary for maintaining and increasing the size of the myofibrils.

Imposing a significant burden to the plantaris by agonist ablation, a higher expression of Yap was found together with a higher transcriptional activity of TEADs into the plantaris, thereby highlighting Yap sensitivity to mechanical loading on the muscle (Goodman et al., 2015). This phenomenon was accompanied by an increase in Akt phosphorylation in a similar pattern as Yap expression and by hypertrophy that persisted following inhibition of mTOR by rapamycin injection. Corroborating previous results (Wang et al., 2015b), it seems that Yap can induce hypertrophy independently of mTOR. This hypertrophy was probably due, at least in part, to a low expression of MuRF-1 and a high expression of MyoD and c-Myc (Goodman et al., 2015).

It therefore appears that Hippo interacts with other pathways for the management of muscle mass. While some studies suggest that Yap overexpression/activation induces muscle atrophy (Judson et al., 2013), others found opposite results, i.e. hypertrophy after Yap overexpression (Goodman et al., 2015; Watt et al., 2015). Those discrepancies can, at least partially, be explained by the different ways, viral vectors vs. gene mutations, used to induce Yap expression and the duration of the treatment (Watt et al., 2010, 2015; Judson et al., 2013; Wei et al., 2013; Goodman et al., 2015). But other unknown factors are probably involved as well. This topic definitely deserves further investigation.

## Perspectives and conclusion

Only one study has investigated the effect of exercise on the Hippo pathway (Hulmi et al., 2013). This study was performed in mice and the chosen exercise paradigm was running, which is not expected to increase muscle mass contrary to resistance exercise. Based on its role in skeletal muscle and satellite cells, we hypothesize that the Hippo pathway could contribute to the accretion of muscle mass after resistance exercise training, which still remains to be tested. In this perspective, the next step is to test whether the results obtained *in vitro* and in mice can be confirmed in healthy human skeletal muscle. Due to its role in the management of satellite cells, we speculate that the Hippo pathway could play a determinant role in the activation of this specific cell type after resistance exercise. Finally, it would be interesting to test the involvement of the Hippo pathway after resistance exercise with blood flow restriction. Indeed, this kind of training has proven its efficiency to increase muscle mass by, amongst others, activating satellite cells and stimulating their proliferation (Nielsen et al., 2012). Here as well, it is unknown whether the Hippo pathway mediates this effect.

## Author contributions

OG wrote the first draft of the manuscript and approved the final version. MF and LD corrected the draft and wrote the final version.

### Conflict of interest statement

The authors declare that the research was conducted in the absence of any commercial or financial relationships that could be construed as a potential conflict of interest.
